# A case of duodenal hemorrhage due to arteriovenous malformation around a serous cystic neoplasm

**DOI:** 10.1186/s40792-018-0547-8

**Published:** 2018-12-05

**Authors:** Shunsuke Tamura, Yusuke Yamamoto, Yukiyasu Okamura, Teiichi Sugiura, Takaaki Ito, Ryo Ashida, Katsuhisa Ohgi, Nobuyuki Watanabe, Keiko Sasaki, Takashi Sugino, Katsuhiko Uesaka

**Affiliations:** 1 Department of Hepato-Biliary-Pancreatic Surgery, Shizuoka Cancer Center, 1007, Shimo-Nagakubo, Sunto-Nagaizumi, Shizuoka 4118777 Japan; 2 Department of Pathology, Shizuoka Cancer Center, 1007, Shimo-Nagakubo, Sunto-Nagaizumi, Shizuoka 4118777 Japan

**Keywords:** Serous cystic neoplasm, Arteriovenous malformation, Pancreas, Acquired, Vascular endothelial growth factor

## Abstract

**Background:**

No reports have so far described arteriovenous malformation (AVM) in the pancreas caused by a tumor. We herein report a case of pancreatoduodenectomy for a patient who developed duodenal hemorrhage due to AVM developed around serous cystic neoplasm (SCN) of the pancreas.

**Case presentation:**

A 79-year-old man was referred to our hospital because of anemia (Hb 7.4 g/dl) and pancreatic head tumor. Computed tomography showed microcystic-type SCN, 87 mm in size, in the pancreatic head. Vascular hyperplasia had developed around the cystic lesion. Upper gastrointestinal endoscopy and colonoscopy did not reveal the cause of anemia, so the patient was followed closely without hemostatic therapy. Iron preparations had improved the anemia. Three months later, the patient developed anemia (Hb 5.8 g/dl) again. Gastrointestinal endoscopy showed oozing from the mucosa in the duodenum via the swollen vascular hyperplasia. He was diagnosed as duodenal hemorrhage from the blood vessels around SCN. Pancreatoduodenectomy was performed to control repeated duodenal bleeding. A histopathological examination revealed that the cystic lesion in the pancreatic head was SCN, and the AVM developed around SCN and duodenum, causing repeated duodenal hemorrhage. The patient was discharged on postoperative day 22. Nine months after surgery, the patient had no recurrence of anemia.

**Conclusions:**

There have been no reports of duodenal hemorrhage due to acquired pancreatic AVM around pancreatic tumor, including SCN. We successfully treated a case of duodenal hemorrhage due to pancreatic AVM around SCN by pancreatoduodenectomy.

## Background

Serous cystic neoplasm (SCN) constitutes only 1–2% of pancreatic neoplasms and 10–15% of all cystic masses of the pancreas [1, 2]. SCN of the pancreas is regarded as a benign entity with rare malignant potential. SCN has hypervascularity and sometimes induces intratumoral hemorrhage [3, 4]. However, only a few cases of SCN inducing gastrointestinal bleeding or hemoperitoneum have been reported [5–9].

Arteriovenous malformation (AVM) is an aberrant vascular shunt between the arterial and venous systems due to the absence of an intervening capillary bed [10]. Pancreatic AVM is rare, and previous reports have shown that pancreatic AVM account for only 0.9–5% of AVM in the gastrointestinal system [11, 12]. AVM of the pancreas has been reported to occasionally induce gastrointestinal bleeding [13]. The proposed causes of pancreatic AVM include congenital malformation (90%) and acquired causes, such as trauma or inflammation [12]; however, the development of pancreatic AVM around a pancreatic tumor, including SCN, has never been reported.

We herein report a case of AVM that developed around SCN in the pancreatic head and caused duodenal hemorrhage.

## Case presentation

**Fig. 1:**
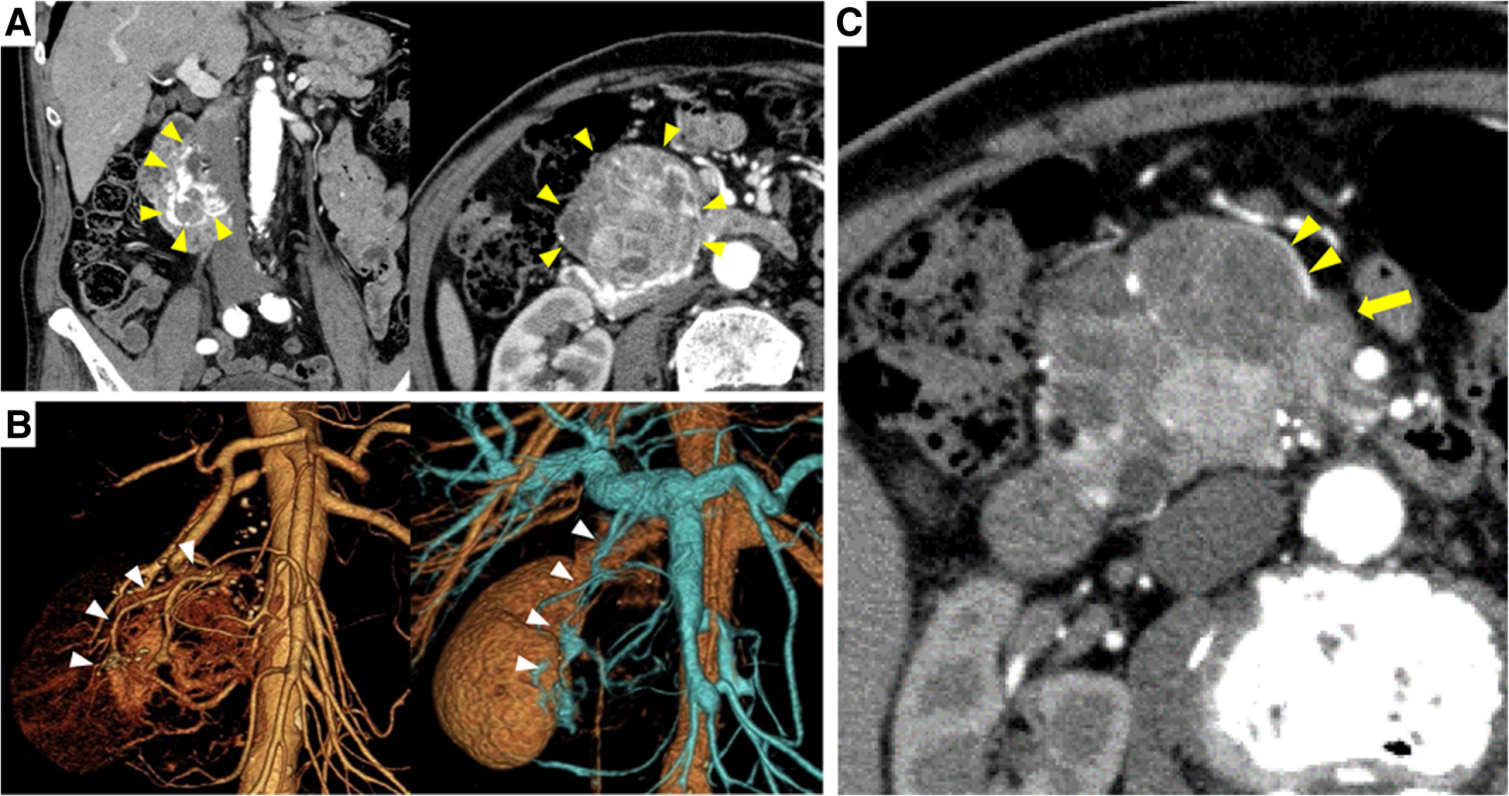
**a** Computed tomography (CT) of the serous cystic neoplasm (SCN) with hypervascularity in the pancreatic head and duodenum (arrow heads) during the late arterial phase. **b** Three-dimensional CT shows abnormal vasculature through the SCN in the arterial and venous phase (arrow heads). **c** The vein of the abnormal vasculature around the SCN was enhanced in the artery phase (arrow heads). The superior mesenteric vein was not enhanced (arrow)

**Fig. 2:**
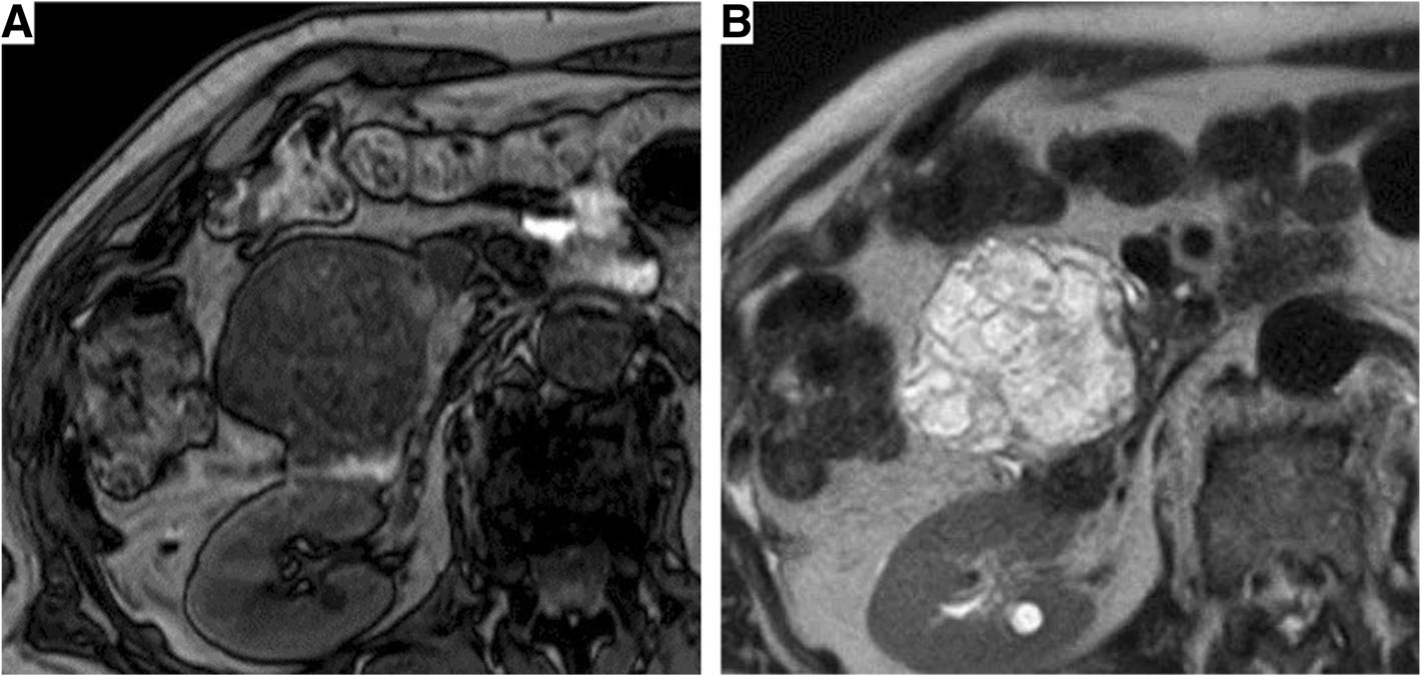
On magnetic resonance imaging, the microcysts were hypointense on T1-weighted imaging (**a**) and hyperintense with the septum on T2-weighted imaging (**b**)

A 79-year-old man who had been suffering from anemia for 7 weeks presented to another local hospital. Because a pancreatic head tumor was detected by computed tomography (CT), he was referred to our hospital for the further examination and treatment. Laboratory tests showed severe anemia with hemoglobin levels of 7.4 g/dl, and tumor markers were within normal ranges (CEA 3.9 ng/mL, CA19–9 24 U/mL). CT showed a microcystic lesion that was enhanced as a honeycomb-like-structure, 87 mm in size, in the pancreatic head. Vascular hyperplasia had developed around the cystic lesion and duodenum (Fig. 1a, b). The artery and vein of the abnormal vessel around the SCN were enhanced in the arterial phase (Fig. 1c). On magnetic resonance imaging, the microcystic lesion was hyperintense on T2-weighted imaging with the septum (Fig. 2a, b), so the lesion was diagnosed as microcystic-type SCN. Upper gastrointestinal endoscopy, capsule endoscope, and colonoscopy failed to detect the cause of anemia, so the patient was followed closely without treatment.

**Fig. 3:**
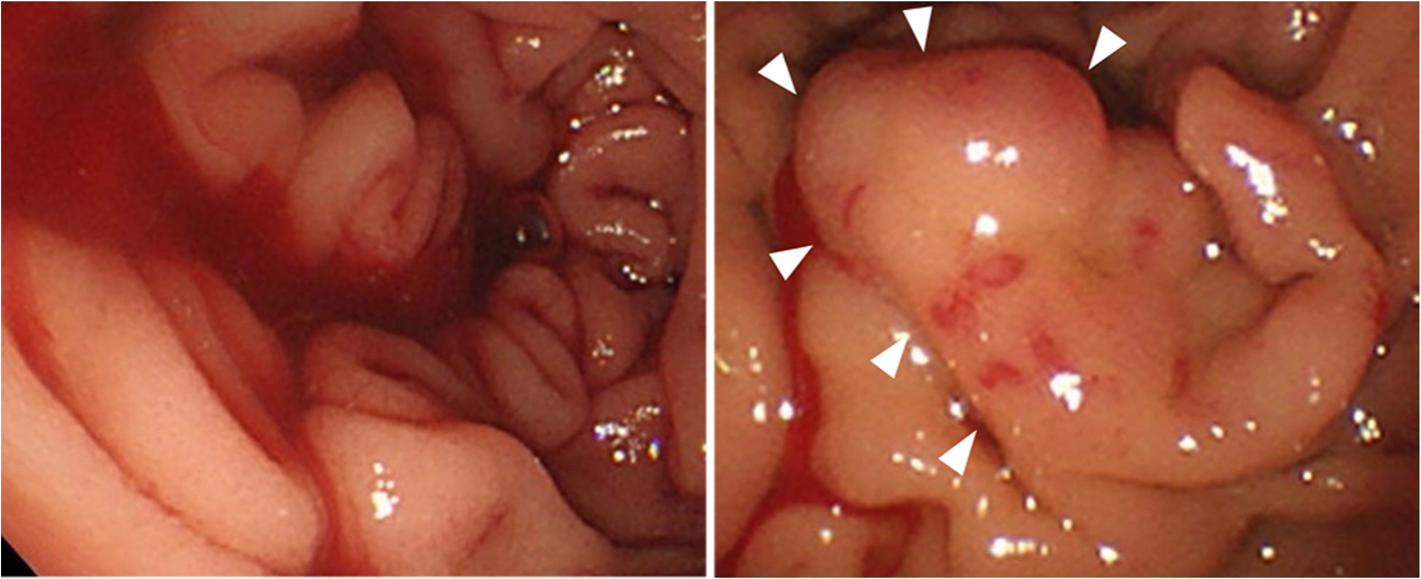
Gastrointestinal endoscopy showed oozing from the swollen vascular hyperplasia (arrow heads) in the duodenum

Three months later, the patient developed anemia (Hb 5.8 g/dl) again. Gastrointestinal endoscopy showed oozing from the mucosa in the duodenum via the swollen vascular hyperplasia (Fig. 3). Duodenal hemorrhage caused by the abnormal vessels around the SCN was highly suspected as the culprit. Therefore, pancreatoduodenectomy was performed. First, the inferior pancreaticoduodenal artery and gastroduodenal artery were divided to control intraoperative bleeding from the abnormal vessels around the SCN. After removing the specimen, reconstruction was performed via the modified Child method. The operative time was 479 min, the intraoperative blood loss was 611 mL, and red blood cell transfusion was performed (560 mL). Postoperatively, the patient developed biochemical leak of pancreatic fistula (the International Study Group of Pancreatic Fistula), but this complication was successfully treated conservatively. The patient was discharged on postoperative day 22.

**Fig. 4:**
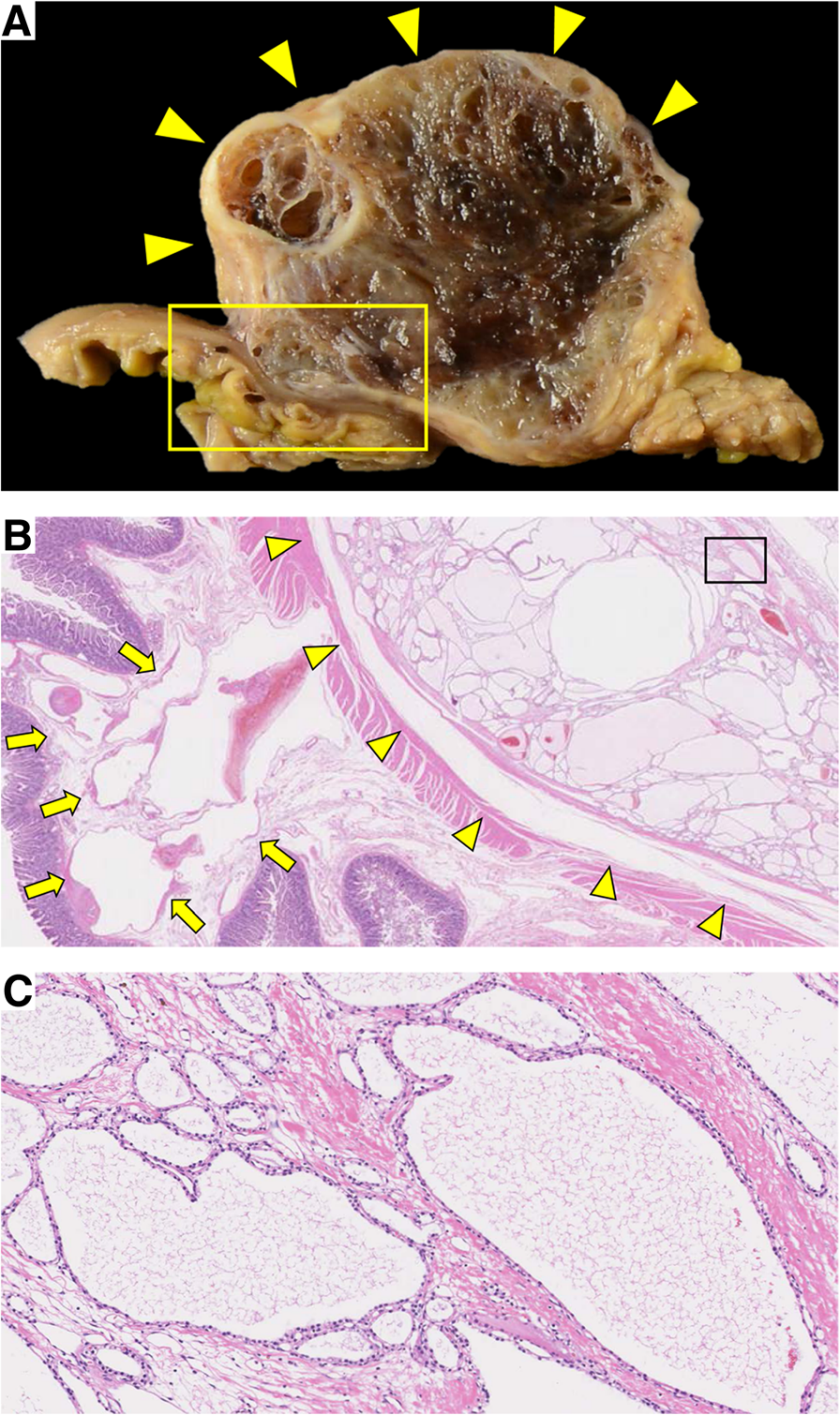
**a** The pancreatic head lesion had innumerable microcysts (arrowheads). **b** Expansion of the square area of (**a**). The border between SCN and duodenum (arrowheads). The abnormal vessels in the duodenal mucosa (arrows) (HE). **c** Expansion of the square area of (**b**). The cyst locules lined by epithelial cells with round central nuclei (HE)

**Fig. 5:**
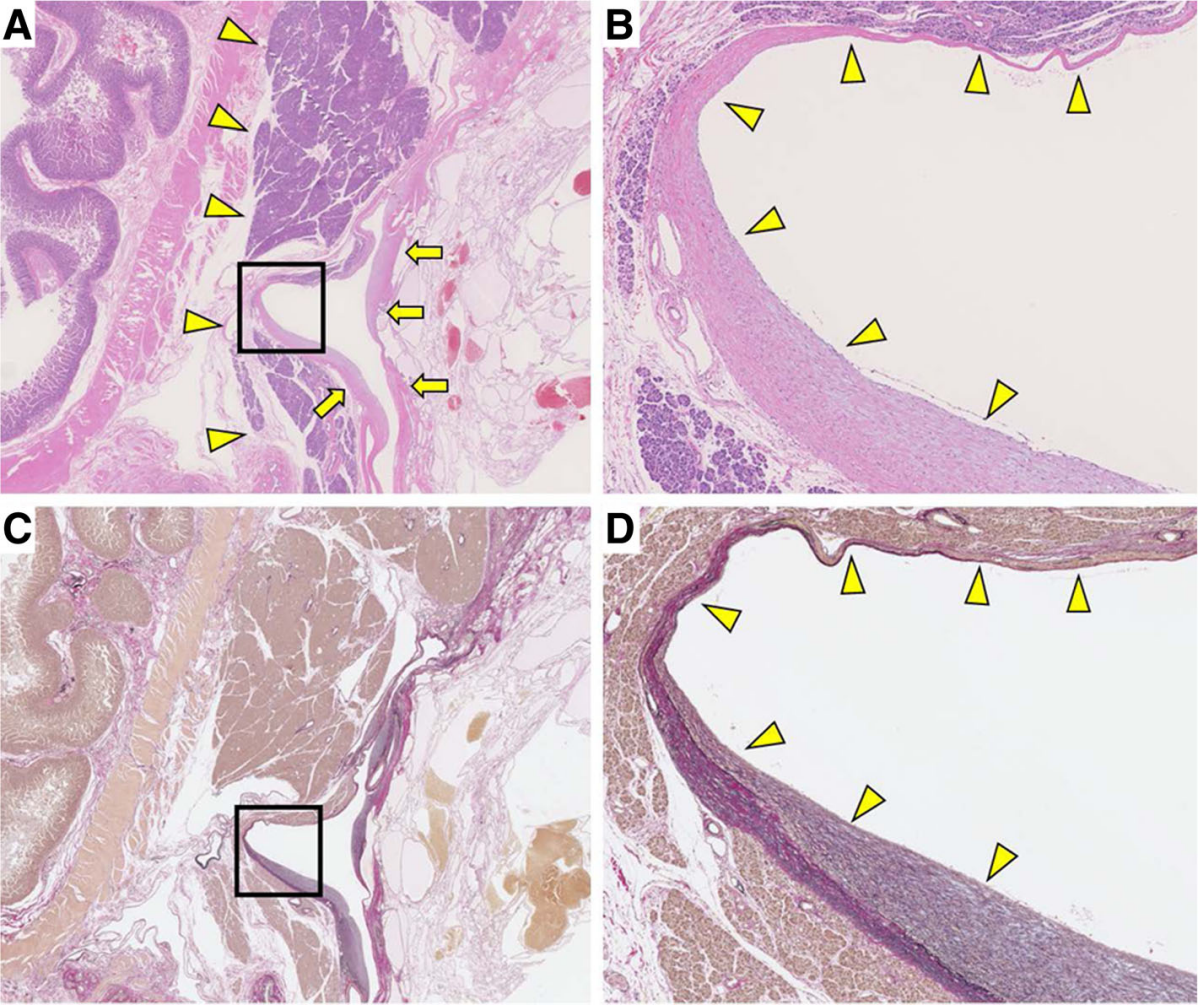
**a** The walls of the abnormal vessels demonstrated both thin and thick walls around the pancreatic SCN (arrows). The border between duodenum and pancreas (arrowheads) (HE). **b** Expansion of the square area of (**a**). The walls of the abnormal vessels demonstrated heterogeneous thickness (arrowheads). **c** The results of Elastica van Gieson staining in the abnormal vasculature. **d** Expansion of the square area of (**c**). The elastic fibers of the abnormal vessels demonstrated heterogeneous thickness (arrowheads)

Macroscopically, the pancreatic head lesion was 90 mm in diameter, circumscribed, and well-demarcated from the surrounding pancreas, and had innumerable microcysts (Fig. 4a). Microscopically, the lesion was composed of cyst locules lined by epithelial cells with round central nuclei (Fig. 4b, c). The lesion was diagnosed as pancreatic SCN. Abnormal vessels had developed around the SCN and duodenum. In the pancreatic SCN and duodenal submucosa, the development of abnormal vessels, which demonstrate a variety of wall thicknesses, was observed. The elastic fibers of these abnormal vessels showed heterogenous thickness and stained positively for Elastica van Gieson staining. In addition, the structure of these abnormal vessels was different from that of the arteries and veins (Figs. 4b and 5a–d). The abnormal vessels were thus diagnosed as being AVM, and this was thought to most likely be the cause of the duodenal hemorrhage observed in this case.

The patient showed no recurrence of anemia or SCN in the 16 months after the operation.

## Discussion

Patients with SCN rarely develop severe symptoms. Jais et al. reported that most patient with SCN were asymptomatic (61%) or had mild symptoms (e.g., abdominal pain, pancreaticobiliary symptoms, diabetes mellitus, abdominal mass, asthenia, nausea, and vomiting) [14]. Intratumoral hemorrhage in SCN occasionally develops [3]. However, gastrointestinal bleeding and hemoperitoneum caused by SCN are rare, and only a few cases of gastrointestinal bleeding or hemoperitoneum caused by rupture of a tumor or duodenal ulcer have been reported (Table 1) [5–9]. Gastrointestinal bleeding due to the development of pancreatic AVM around an SCN has never been reported. To our knowledge, this is the first report of gastrointestinal bleeding due to the development of pancreatic AVM around SCN.

**Table 1:** Previous case reports of GI bleeding or hemoperitoneum due to SCN

No.	Author	Year	Age (years)	Sex	Tumor size (mm)	Type of bleeding	Cause of bleeding	Surgery
1	Rosendaum H [5]	1963	69	M	100	GI bleeding	Rupture of tumor	PD
2	Compagno J [6]	1978	84	F	–	GI bleeding	Duodenal ulcer bleeding	No surgery
3	Pyke CM [7]	1992	–	–	–	Hemoperitoneum	Rupture of tumor	Celiotomy
4	Sakaguchi T [8]	2000	38	M	130	Hemoperitoneum	Rupture of tumor	DP
5	Ashkzaran H [9]	2007	55	M	110	Hemoperitoneum	Rupture of tumor	DP
6	Present case	2018	79	M	90	GI bleeding	Rupture of AVM	PD

A few cases have been reported, and most of cases of bleeding were rupture of tumor

*GI* gastrointestinal, *PD* pancreatoduodenectomy, *DP* distal pancreatectomy, *M* male, *F* female

Pancreatic AVM is defined as a timorous formation or vascular anomaly that builds up via an aberrant bypass anastomosis of the arterial and venous systems in the pancreas [15]. Pancreatic AVM is rare, and previous reports have shown that pancreatic AVM accounts for only 0.9–5% of cases of AVM in the gastrointestinal system [11, 12]. Among patients with pancreatic AVM, 90.5% were thought to have the congenital type, which occurs as part of a broader syndrome, such as Osler Weber Rendu [16]. In contrast, acquired-type AVM is generally caused by trauma, tumor, or inflammation [12]. Regarding acquired AVM caused by a tumor, several reports have described cases of acquired AVM due to renal cell carcinoma (RCC), as RCC sometimes induces the development of AVM in the kidney and elsewhere [17, 18]. However, pancreatic AVM around the pancreatic tumor has never been reported.

In the present case, the histopathological examination revealed that abnormal blood vessels had developed around the SCN and the duodenum. These blood vessels penetrated the SCN. It is important to compare the previous imaging findings of these patients with later scans in order to prove that the pancreatic AVM was the acquired subtype; however, previous scans of the pancreas were unavailable. We believe that the AVM which induced duodenal hemorrhaging might have developed due to SCN, given the clinical course, lack of a family history, and lack of a bleeding tendency. Chou et al. summarized previous cases of pancreatic AVM, and the median age at the diagnosis was 50 years (range 0.6–75 years) [12]. The present patient was 79 years old, falling slightly outside of the range of previous patients with pancreatic AVM.

**Fig. 6:**
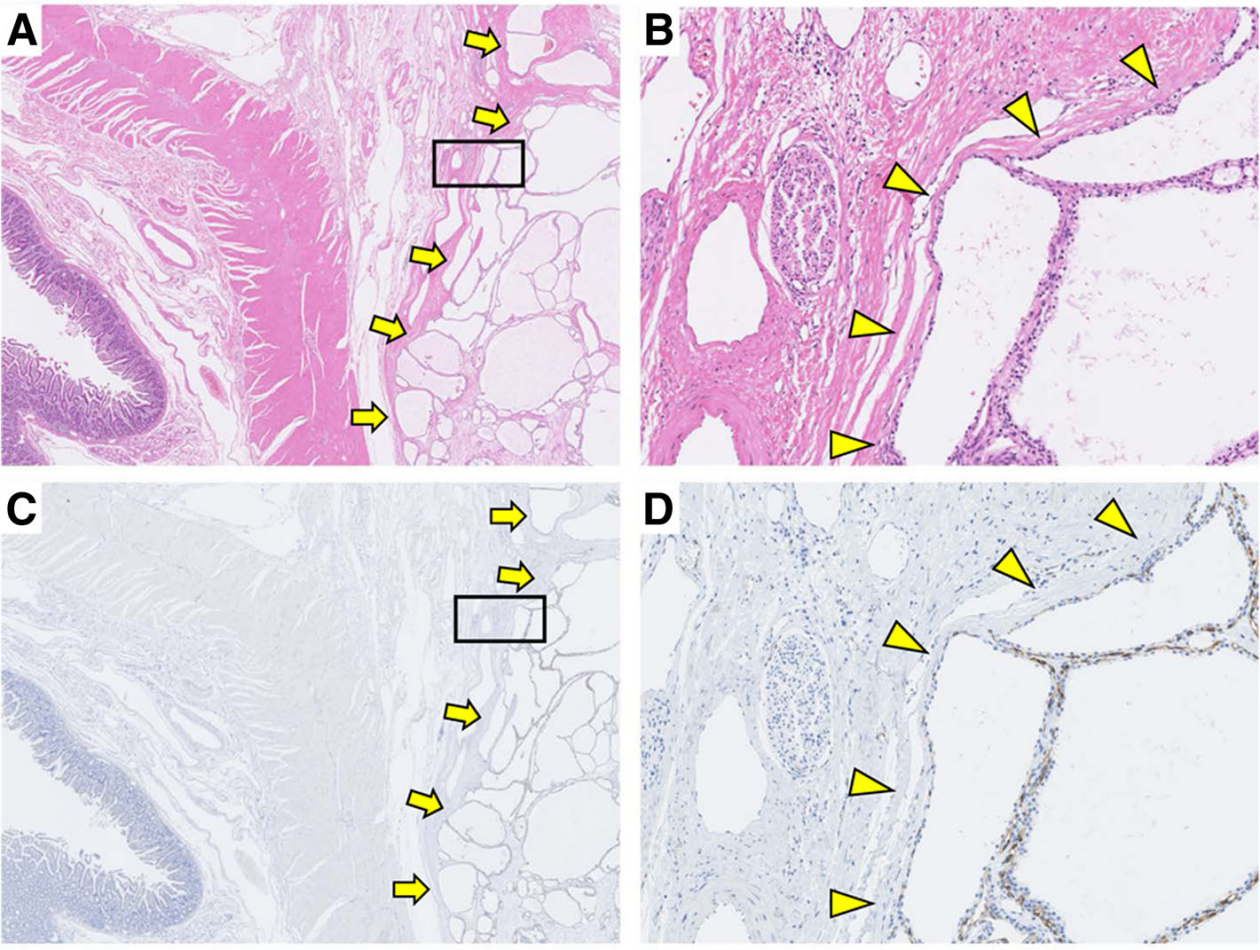
**a** The border between the pancreas and SCN (arrows) (HE). **b** Close-up of the square area of (**a**). The border between the pancreas and SCN (arrowheads) (HE). **c** The VEGFR2-stained vascular tissue around SCN (right-side arrows). The pancreas and duodenum were not stained by VEGFR2 (left-side arrows). **d** Close-up of the square area of (**c**). VEGFR2 specifically stained the vascular region around SCN. The border between the pancreas and SCN (arrowheads)

Several reports have recently addressed the relationship of SCN and vascular endothelial growth factor (VEGF) with hypervascularity, which was specifically elevated in both the SCN cyst fluid and lesion tissue [19, 20]. VEGF is crucial for the development of AVM, and previous reports have shown that high VEGF levels in RCC cause acquired AVM [17, 18]. In the present case, microvascular hyperplasia was detected around SCN, and the AVM surrounded the SCN. Although acquired AVM has been reported to be caused by trauma, inflammation, and tumors [12], present case had no history of trauma. SCN causes slight inflammation but not enough to induce pancreatitis. In the present case, immunohistochemical findings showed that the type 2 VEGF receptor (VEGFR2) was expressed specifically in the endothelium of vascular tissue around SCN (Fig. 6a–d). However, VEGF antibody stains not only the SCN cellular membrane but also the duodenal membrane and normal pancreas. Chris et al. mentioned that some VEGF antibodies were regarded as unfit for VEGF immunostaining based on poor immunostaining criteria, thus making it difficult to accurately stain VEGF [21]. We therefore cannot draw definitive conclusions based on the present immunohistochemical findings. However, the expression of VEGF and VEGFR2 by SCN may have promoted angiogenesis, thereby inducing the development of acquired AVM around pancreatic SCN.

In previous reports, clinical symptoms included gastrointestinal bleeding (49.3%) and abdominal pain and/or back pain (40.6%), while patients (18.8%) were asymptomatic [22]. The causes of gastrointestinal bleeding from pancreatic AVM in 26 patients were reported. Esophageal or gastric varices from portal hypertension were observed in 11 patients (42%). Duodenal ulcer or duodenal bleeding was observed in 13 patients (50%). Hemobilia was observed in three patients (12%), and pancreatic duct bleeding was observed in two patients (8%) [13]. The treatment of pancreatic AVM is controversial. In pancreatic AVM case series reported by Chou et al., surgical resection has been considered the first choice (43.8%), but it is not always feasible given its invasiveness. Transarterial embolization (TAE) alone (11.2%), TAE + surgery (10.1%), and radiotherapy (2.2%) have been attempted as alternative or adjunct treatments [12]. Some reports recommend surgical resection or TAE for not only symptomatic pancreatic AVM but also asymptomatic cases due to the risk of gastrointestinal bleeding or rupture of the esophageal varices in response to the portal hypertension induced by pancreatic AVM [12, 22]. We decided to perform pancreatoduodenectomy in the present case to control repeated duodenal bleeding caused by the AVM that had developed around the SCN. Pancreatectomy should be considered in patients who develop gastrointestinal bleeding from pancreatic AVM around a pancreatic tumor, as it can be difficult to control the duodenal bleeding by other treatments.

## Conclusion

There have been no reports of duodenal hemorrhage due to acquired pancreatic AVM around pancreatic tumor. We successfully treated duodenal hemorrhage due to pancreatic AVM around SCN by pancreatoduodenectomy.

## Abbreviations

AVM: Arteriovenous malformation
CT: Computed tomography
RCC: Renal cell carcinoma
SCN: Serous cystic neoplasm
TAE: Transarterial embolization
VEGF: Vascular endothelial growth factor
VEGFR2: Type 2 vascular endothelial growth factor receptor

## References

[1] Horvath KD, Chabot JA (1999). An aggressive resectional approach to cystic neoplasms of the pancreas. Am J Surg.

[2] Winter JM, Cameron JL, Lillemoe KD, Campbell KA, Chang D, Riall TS (2006). Periampullary and pancreatic incidentaloma: a single institution’s experience with an increasingly common diagnosis. Ann Surg.

[3] Choi JY, Kim MJ, Lee JY, Lim JS, Chung JJ, Kim KW (2009). Typical and atypical manifestations of serous cystadenoma of the pancreas: imaging findings with pathologic correlation. AJR Am J Roentgenol.

[4] Sumiyoshi T, Shima Y, Okabayashi T, Kozuki A, Nakamura T, Iwata J (2015). Rapid shrinkage of a pancreatic serous cystadenoma with cystic degeneration: report of a case. Surg Today.

[5] Rosenbaum H, Connolly PJ, Climie AR, Reveno WS (1963). Pancreatic cystadenoma with intestinal hemorrhage. Am J Roentgenol Radium Therapy, Nucl Med.

[6] Compagno J, Oertel JE (1978). Microcystic adenomas of the pancreas (glycogen-rich cystadenomas): a clinicopathologic study of 34 cases. Am J Clin Pathol.

[7] Pyke CM, van Heerden JA, Colby TV, Sarr MG, Weaver AL (1992). The spectrum of serous cystadenoma of the pancreas. Clinical, pathologic, and surgical aspects. Ann Surg.

[8] Sakaguchi T, Nakamura S, Suzuki S, Konno H, Fujita K, Suzuki K (2000). Intracystic hemorrhage of pancreatic serous cystadenoma after renal transplantation: report of a case. Surg Today.

[9] Ashkzaran H, Coenegrachts K, Steyaert L, Vandelanotte M, van den Berghe I, Verstraete K (2007). An unusual presentation of pancreatic serous cystadenoma with acute hemorrhage. JBR-BTR.

[10] Fleetwood IG, Steinberg GK (2002). Arteriovenous malformations. Lancet.

[11] Meyer CT, Troncale FJ, Galloway S, Sheahan DG (1981). Arteriovenous malformations of the bowel: an analysis of 22 cases and a review of the literature. Medicine (Baltimore).

[12] Chou SC, Shyr YM, Wang SE (2013). Pancreatic arteriovenous malformation. J Gastrointest Surg.

[13] Hosogi H, Ikai I, Hatano E, Taura K, Fujii H, Yamamoto Y (2006). Pancreatic arteriovenous malformation with portal hypertension. J Hepato-Biliary-Pancreat Surg.

[14] Jais B, Rebours V, Malleo G, Salvia R, Fontana M, Maggino L (2016). Serous cystic neoplasm of the pancreas: a multinational study of 2622 patients under the auspices of the International Association of Pancreatology and European Pancreatic Club (European study group on cystic tumors of the pancreas). Gut.

[15] Halpern M, Turner AF, Citron BP (1968). Hereditary hemorrhagic telangiectasia. An angiographic study of abdominal visceral angiodysplasias associated with gastrointestinal hemorrhage. Radiology.

[16] Nassiri N, Cirillo-Penn NC, Thomas J (2015). Evaluation and management of congenital peripheral arteriovenous malformations. J Vasc Surg.

[17] Volin S, Steinberg P, Mittleider D (2013). Renal cell carcinoma initially presenting as an arteriovenous malformation: a case presentation and a review of the literature. Case Rep Urol.

[18] Albandar HJ, Roberto ES, See JRH, Sabiers JH (2017). Arteriovenous malformation and thyroid metastasis from underlying renal cell carcinoma, an unusual presentation of malignancy: a case report. Oncol Lett.

[19] Yip-Schneider MT, Wu H, Dumas RP, Hancock BA, Agaram N, Radovich M (2014). Vascular endothelial growth factor, a novel and highly accurate pancreatic fluid biomarker for serous pancreatic cysts. J Am Coll Surg.

[20] Carr Rosalie A., Yip-Schneider Michele T., Dolejs Scott, Hancock Bradley A., Wu Huangbing, Radovich Milan, Schmidt C. Max (2017). Pancreatic Cyst Fluid Vascular Endothelial Growth Factor A and Carcinoembryonic Antigen: A Highly Accurate Test for the Diagnosis of Serous Cystic Neoplasm. Journal of the American College of Surgeons.

[21] van der Loos CM, Meijer-Jorna LB, Broekmans ME, Ploegmakers HP, Teeling P, de Boer OJ (2010). Anti-human vascular endothelial growth factor (VEGF) antibody selection for immunohistochemical staining of proliferating blood vessels. J Histochem Cytochem.

[22] Song KB, Kim SC, Park JB, Kim YH, Jung YS, Kim MH (2012). Surgical outcomes of pancreatic arteriovenous malformation in a single center and review of literature. Pancreas.

